# Home Blood Pressure Management Intervention in Low- to Middle-Income Countries: Protocol for a Mixed Methods Study

**DOI:** 10.2196/resprot.7148

**Published:** 2017-10-16

**Authors:** Cheryl Anderson, Sufia Dadabhai, Albertino Damasceno, Anastase Dzudie, Sheikh Mohammed Shariful Islam, Deepak Kamath, Namratha Kandula, Noel Kayange, Renato Quispe, Ambuj Roy, Syed Shah, Rafael Vidal-Perez

**Affiliations:** ^1^ Division of Preventive Medicine Department of Family Medicine and Public Health University of California San Diego La Jolla, CA United States; ^2^ Infectious Disease Epidemiology Division Department of Epidemiology Johns Hopkins University Baltimore, MD United States; ^3^ Department of Cardiology, Clinical Trials, Epidemiology Eduardo Mondlane University Maputo Mozambique; ^4^ Cardiology, Internal Medicine Hôpital Général de Douala Douala Cameroon; ^5^ International Centre for Diarrhoeal Disease Research Dhaka Bangladesh; ^6^ Clinical Research and Training Department of Pharmacology & Division of Clinical Research and Training St. John's Research Institute Karnataka India; ^7^ Internal Medicine Northwestern Medical Group Chicago, IL United States; ^8^ Internal Medicine (General Medicine), Infectious Diseases, Cardiology London School of Hygiene and Tropical Medicine London United Kingdom; ^9^ Universidad Peruana Cayetano Heredia Lima Peru; ^10^ Department of Cardiology Emory University Atlanta, GA United States; ^11^ College of Medicine and Health Sciences United Arab Emirates University Al Ain United Arab Emirates; ^12^ Department of Cardiology Hospital Universitario Lucus Augusti Lugo Spain

**Keywords:** blood pressure, home management, self-management, LMIC

## Abstract

**Background:**

Control of hypertension in low- and middle-income countries (LMICs) is poor, often less than 10%. A strong body of evidence demonstrates that home blood pressure management lowers blood pressure, and recent guidelines from the National Institute for Clinical Health and Excellence recommends home blood pressure monitoring. However, the preponderance of data on the benefits of home blood pressure management comes from studies in high-income countries.

**Objective:**

The objective of the study is to examine whether an intervention of home blood pressure management is feasible in LMICs. Home blood pressure management is defined as self-monitoring of blood pressure and self-titration of antihypertensive medications. We will identify barriers and facilitators of home blood pressure management and explore unique contextual factors in LMICs that influence implementation of home blood pressure management.

**Methods:**

Participants will be recruited from 6 sites from 2015 to 2018. Patients and health care workers will be included. We will use mixed methods including focus groups, interviews, and standardized checklists. When possible, we will adapt materials from prior successful studies so that they are culturally and contextually appropriate.

**Results:**

This ongoing study is funded by the World Heart Federation. The information that is obtained will be used to develop a randomized clinical trial of home blood pressure management in LMICs.

**Conclusions:**

The data generated from this qualitative study will provide much needed information from patients and health care workers about barriers and facilitators of home blood pressure management and unique contextual factors that might influence implementation of home blood pressure management in LMICs.

## Introduction

### Background

Control of hypertension in low- and middle-income countries (LMICs) is poor [1]. It has been documented that home blood pressure (BP) management significantly lowers BP when compared to usual care in high-income countries [2-6]. One study of home BP management in a high-income country (N=527) showed, at 6 months after intervention, a decrease in systolic blood pressure (SBP) of 12.9 mm Hg (95% CI 10.4 to 15.5) in the self-management group versus 9.2 mm Hg (95% CI 6.7 to 11.8) in the control group. The decrease in SBP was even higher at 12 months after intervention: 17.6 mm Hg (95% CI 14.9 to 20.3) and 12.2 mm Hg (95% CI 9.4 to 14.9), respectively [3]. This strategy for BP management has been incorporated into the National Institute for Health and Care Excellence guidelines in the United Kingdom [7]. As such, our research question is relevant to public health efforts and clinical practice.

Recent data suggest that the utility of home BP monitors may be limited in resource-restricted settings in the United States [8]. However, there are little data from LMICs [9]. One recent clinical trial in Mexico and Honduras documented that individuals using a BP monitor combined with automated interactive voice response messages had SBP levels 4.2 mm Hg (95% CI –9.1 to 0.7; *P*=.09) lower on average than control group. Furthermore, a subgroup of individuals with low literacy showed a higher decrease, 8.8 mm Hg (95% CI –14.2 to –3.4; *P*=.002). However, this study had important limitations such as short duration of follow-up, small sample (N=181), and limited interface with the health care system [9].

**Figure 1:**
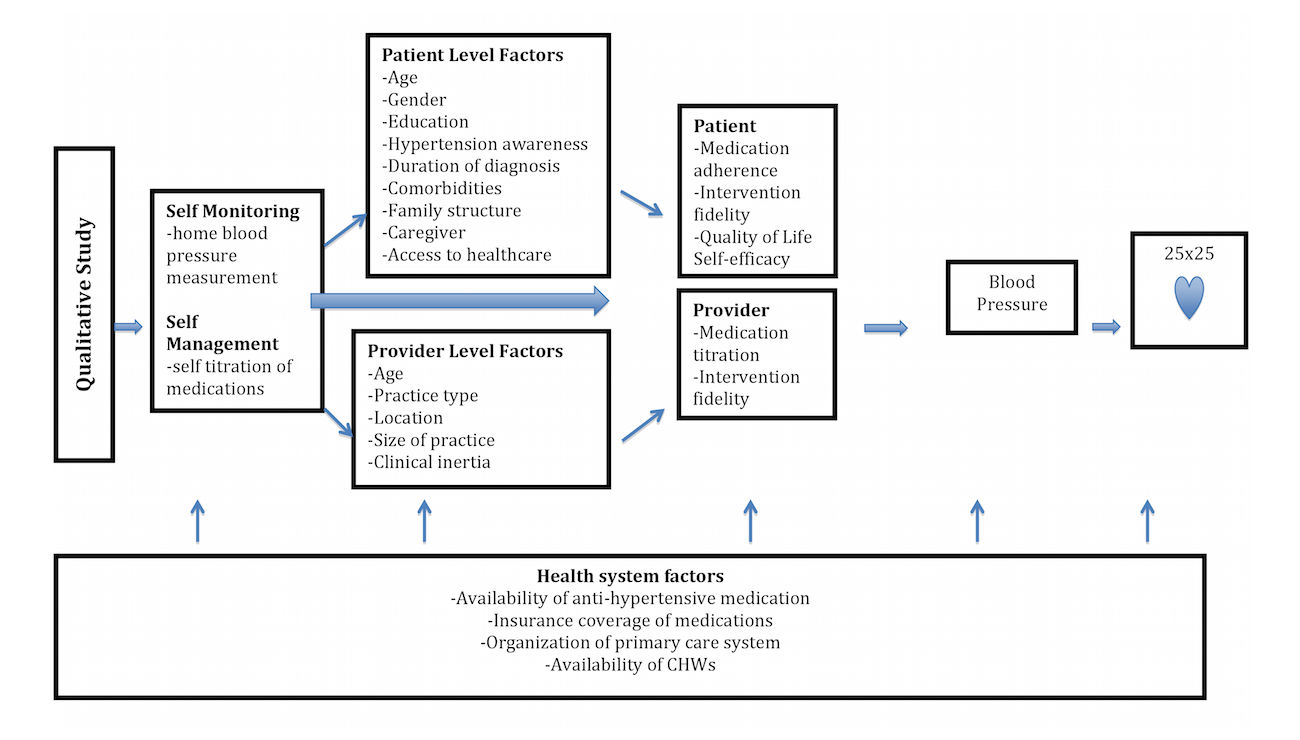
Conceptual framework describing the relationship between home blood pressure management context and blood pressure.

The potential impact of this work is high because of the high global burden of hypertension in LMICs and the double burden of disease in health care systems of these countries; therefore, identification of innovative and effective strategies to control BP is critical. To the best of our knowledge, our study aims to be one of the first to provide evidence of the feasibility and acceptability of home BP management in these settings. Figure 1 shows our conceptual framework for the relationships between home BP management context and BP. We hypothesize that self-management will optimize management of BP in a low-income setting by reducing delays in medication titration and improving adherence to antihypertensive medication.

The overall objective of this project is to examine whether an intervention of home BP management, without telemonitoring, is feasible in LMICs. Given that this project will be conducted in LMICs, we will not use home BP telemonitoring technologies. For home monitoring of BP, we will use the M6 Comfort Blood Pressure Monitor (Omron Healthcare) device. To our knowledge, the M6 Comfort was not developed with consideration for challenges with power supply and, to address this, we provided a fresh set of batteries each time a new participant used the BP monitor. Of note, the BP monitors and batteries used at the Malawi site were purchased in South Africa, because quality of local batteries is a challenge and the Omron brand is not readily available locally.

There are important implications of this work regardless of the findings. The data generated will provide much needed information from patients about barriers and facilitators of home BP management. We will learn about challenges patients may have with using a home BP monitor in an LMIC setting, role of caregivers in home monitoring, and general interest in a clinical trial that we hope to conduct regarding home management of BP. Additionally, we will obtain information on health care provider perspectives on intervention feasibility and acceptability and possible importance of medication dose titration as a component of self-management of BP. The findings from this qualitative study will guide the development and refinement of a clinical trial of home BP management in LMICs.

### Aims and Hypotheses

Aim #1: To assess the barriers, facilitators, and context for home BP management in LMICs.

Hypothesis A: There will be unique contextual patient-level factors such as hypertension awareness, duration of diagnosis, comorbidities, availability of a caregiver, patient-provider relationship, and access to health care that will inform the design of a clinical trial of home BP management in LMICs.

Hypothesis B: There will be unique contextual provider-level factors such as location and size of practice, patient-provider relationship, and comfort with home medication titration protocol that will inform the design of a clinical trial of home BP management in LMICs.

Aim #2: To conduct a process evaluation to explore intervention feasibility and acceptability.

Aim #3: To beta-test home BP monitors and titration protocols and materials.

## Methods

### Human Subjects

To ensure the protection of human subjects, we obtained ethical clearance at all participating sites. Institutional Review Board applications were submitted prior to beginning all work.

### Informed Consent

Written informed consent will be obtained from study participants prior to any study activities involving data collection.

### Study Population

#### Aim 1

To address aim 1, we will obtain information from patients, family caregivers, and health care providers. The goal is to understand the barriers and facilitators of self-monitoring BP and medication self-titration in LMICs. We will recruit 24 to 36 patients, family caregivers, and stakeholders at each of the 6 sites: Gilgit, Pakistan; Blantyre, Malawi; Bangalore, India; Dhaka, Bangladesh; Lima, Peru; and Douala, Cameroon. Countries were selected based on the availability of research partners with interest and capacity to conduct this work. Sampling will be purposive and heterogeneous to capture individuals with family caregivers and those without and those with longer standing hypertension (≥3 years) and those with a newer diagnosis (<3 years). However, we will exclude participants with resistant hypertension. Study participants will be selected through outpatient medical clinics in each city. Patients from the practices that meet the inclusion criteria will be invited to participate in the study. Key informants (ie, health care providers) from the health care teams and health care systems will be invited through local contacts.

#### Aim 2

For aim 2, we will recruit 8 to 10 health care providers at each of the 6 study sites. The goal is to understand the feasibility and acceptability of self-monitoring BP and medication self-titration from the perspective of health care providers in LMICs. We will recruit a purposive sample of participants with a goal of obtaining equal numbers of men and women. We will also consider age group, type of provider (eg, physician, nurse, community health worker), specialty, and location of practice (ensure that this is complementary with where intervention trial activities may occur at a later date). The sample size of the focus groups with health care providers will be evaluated by study investigators for thematic saturation and redundancy. If new issues emerge, we will enroll additional providers, but if there is thematic saturation and redundancy no additional interviews will be conducted.

#### Aim 3

For aim 3, we will choose 5 patients (some of them will have a family caregiver) at the trial site who meet study inclusion criteria. We will seek the assistance of clinic staff in identifying patients and their caregivers. The sample size for this aim will be evaluated by study investigators for thematic saturation and redundancy. If new issues emerge for the patients as they do the beta-testing, we will enroll additional patients to better understand these issues. However, if there is redundancy in the information we receive, no additional patients will be included. Patients will be asked to use the BP monitors and diaries and provide feedback. We will also recruit providers who would likely participate in our future intervention study about self-management of BP. This will include 10 physicians, nurses, and community health workers. They will be asked to review the study’s medication titration protocol and provide feedback.

### Procedures

#### Aim 1

Perspectives of patients, family caregivers, and health care workers will be obtained using 2 qualitative research techniques: focus group discussions (patients and family caregiver) and in depth semistructured interviews (health workers). At least 2 focus groups of patients with their family caregivers will be formed in each city with 6 to 9 participants in each group. The sample size of the focus groups will be evaluated by study investigators for thematic saturation and redundancy. If new issues emerge we will enroll additional participants, but if there is thematic saturation and redundancy no additional interviews will be conducted. The focus group discussion guide was developed collaboratively using literature review and expert and local team input. Key informant interviews will use a semistructured interview guide developed by the same process. Key informants from the health care systems will include primary care physicians and cardiologists, nurses, and community health workers.

Data collection and data analysis guides and protocols will be standardized across sites to ensure cross-national comparisons of qualitative data, and they will be adapted so that they are culturally and contextually appropriate. We will also use standardized checklists and interviews to obtain information from study participants, and data collection will be conducted by individuals trained in qualitative research methods. The broad themes that will be examined in our qualitative study are summarized in Figure 1, and the interview guides are shown in Multimedia Appendices 1 and . The themes include barriers and facilitators to home BP management in LMICs. We are interested in factors that may occur at the individual level and at the provider level. The individual-level factors are shown in Figure 1 and include age, gender, education, awareness of hypertension, duration of hypertension diagnosis, comorbidities, family structure, caregiver, and access to health care. Provider-level factors are also shown in Figure 1 and include age of the provider, practice type, location of practice, clinical inertia, and size of practice.

The outcomes of interest for the qualitative data examined in aim 1 are barriers, facilitators, and context for home BP management.

#### Aim 2

A semistructured interview will be conducted with health care providers at each site. The interview will cover the following topics: overall experience and attitude about high BP, whether patients are currently asked to measure and monitor their own BP, what are possible barriers to treatment and adherence to BP management strategies, role of the health care provider in helping patients with high BP management, whether they would be interested in participating in an intervention about self-management of BP, and general thoughts about strategies to improve the lives of patients and the treatment of high BP. We will also obtain feedback from providers about the approach that would be used by the patient for self-titration of medication. We are interested in knowing about the following topics: the appropriateness of the frequency of medication titration, clarity and readability of instructions, comfort level of the health care team with the actions recommended, and what can be done to improve successful use of the self-titration protocol by patients.

The outcomes of interest for the qualitative data examined for aim 2 are the perspectives of the health care providers on intervention feasibility and acceptability.

#### Aim 3

The procedures to beta-test the home BP management approach involve 4 steps.

First, patients and caregivers will be trained by study personnel and instructed to use the BP monitor and to record readings in a diary. The patient will perform a BP measurement in front of study staff. The study staff will use predetermined criteria to assess adequacy of training. The patient will be retrained immediately if required. If despite 2 attempts during training the patient is unable to correctly measure his or her BP, a caregiver will be asked to measure the BP. If the caregiver also fails after 2 attempts, the patient and caregiver will be excluded from the study.

Second, study personnel will provide patients with a BP monitor and diary to use at home. Consistent with the protocol for the recently completed highly successful TASMIN trial, patients will be asked to measure their BP 2 times a day over 1 week: once in the morning and once in the evening. They will be required to take 2 readings 5 minutes apart at each time point and record the values in the study diary.

Third, at the end of the week, patients will be asked to return to the clinic for an interview about their experience and how it could be made better and to complete an interviewer-administered usability scale. The interviewer will ask patients for feedback on the following topics: overall experience and attitude towards the monitor, whether they were able to successfully monitor their own BP, what are possible barriers to monitoring their own BP, role of the caregiver in helping to monitor their BP management, what could be done to improve their experience with monitoring their BP at home, and whether they would be interested in using a monitor if selected to do so for the future trial. We will also ask about process variables including whether the patient read and understood the BP diary, the number of BP readings that are stored in the monitor; and the number of BP readings that are recorded in the diaries. The usability scale captures information on multiple aspects of using the BP monitor (eg, ability to understand BP readings, comfort of cuff).

Last, while patients are at the clinic, we will also show them a hypothetical medication up-titration protocol based on their current hypertension medication regimen. This hypothetical protocol will have been designed and approved by a study physician in consultation with the treating physician. Patients will be asked for feedback on barriers and facilitators that may affect fidelity to the hypothetical protocol. We are specifically interested in the clarity and readability of instructions and comfort level of patient and family caregiver with the actions recommended.

The outcomes of interest for the qualitative data examined for aim 3 are patient success with using the home BP monitor, barriers to monitoring BP at home, role of caregiver in home monitoring, interest in a clinical trial about home management of BP, patient understanding of the daily BP diary, number of BP readings taken at home, usability of home BP monitor, clarity of instructions for using the home BP monitor, and fidelity to a hypothetical medication titration protocol.

### Data Management and Analysis

We have guidelines for the number of participants that will be recruited at each site for the study. However, the final sample size for interviews and focus group discussions will be determined based on the principle of saturation. Once the interviews and group discussions begin, the researchers will determine based on the information obtained whether involving another participant or group will add important new data. If no important new data will be added, no additional interviews, focus groups, or beta-testing will be conducted.

All interviews and group discussions will be digitally recorded upon receiving the consent of participants. Recorded interviews and focus group discussions will be reviewed by the local researcher in order to extract the relevant data for analysis. Using the coding on the spot method, each researcher will record the data in the original language in a common template. Data matrices will be then translated into English. The coding on the spot method consists of dividing the interviews (or focus group discussions) into 1-minute segments. The recording is played back for exactly 1 minute at a time, then it is paused and the researcher summarizes what has been said in that minute in the assigned box. If the researcher finds a quote that condenses the main views expressed by the participants, this quote will be transcribed verbatim in the quote box in the language in which it was originally recorded. It is possible that the researcher will not find any useful quotes. If there is an observation that the researcher considers useful for data analysis it will be noted. After completing each recording, the researcher will verify the information entered in the matrix to ensure it is clear and nonredundant. It is also possible that a new topic emerges from the data extraction process, and it is possible for this new topic to be added. Relevant data is that which provides information around the core 7 research topics: (1) BP management and measurement, (2) medication adherence and medication titration: barriers and facilitators, (3) patient-physician relationship, (4) characteristics of the local health care system, (5) perceptions about home BP monitoring, (6) understanding of BP measurements, and (7) social support.

Interim data analysis will occur alongside the data collection. After each data collection round (interview or focus group discussion), the data will be reviewed by the research team to further refine the data collection processes. Data will be analyzed across different languages. Coding and categorization will be carried out by at least 2 team members trained in qualitative methods. Reports of the analysis will be produced for each site for review of local team members. The analysis will use both inductive codes and a priori categories. Final collation of the analyses using the English translations will be centralized for the final analysis. All data from beta-testing of the home BP monitors and titration protocols will be used in an iterative process to adapt and improve BP monitoring and titration materials and protocols.

This study will be conducted over a 30-month period (see Table 1). In addition to obtaining Institutional Review Board approval, study activities will include translation of study material, training of data collectors, implementation of mixed methods, data analysis, and manuscript writing.

**Table 1:** Timeline of activities.

	Months									
Timeline (2015-2016)	1	3	6	9	12	15	18	21	24	27
Obtain Institutional Review Board approval	x	x	x							
Translate study materials	x	x	x							
Train data collectors		x	x	x						
Conduct focus groups			x	x	x	x	x	x	x	
Analyze data					x	x	x	x	x	
Write manuscript						x	x	x	x	x

## Results

This protocol refers to an ongoing study funded by the World Heart Federation. The standardized protocol is being implemented in 6 different LMICs. The information that is obtained will be used to develop a randomized clinical trial of home BP management in LMICs. We expect that there will be unique contextual factors that need to be accounted for before the highly successful interventions from high-income countries can be used.

## Discussion

The rationale for this study is based on (1) documentation that control of hypertension in LMICs is poor, often less than 10%, (2) a strong body of evidence that home BP management lowers blood pressure, (3) recent guidelines from the National Institute for Clinical Health and Excellence that recommend home BP monitoring, and (4) the observation that the preponderance of data on the benefits of home BP management comes from studies in high-income countries.

The data generated from this qualitative study will provide much needed information from patients and health care workers about barriers and facilitators of home BP management and unique contextual factors that might influence implementation of home BP management in LMICs. A clinical trial of home BP management is needed to quantify the potential benefits in this setting.

## Appendix

Multimedia Appendix 1 Semistructured interview guide for patients and family caregivers.

Multimedia Appendix 2 Semistructured interview guide for health care providers.

## Abbreviations

BP: blood pressure
LMIC: low- and middle-income country
SBP: systolic blood pressure
