# Supplementary material for: Home Blood Pressure Management Intervention in Low- to Middle-Income Countries: Protocol for a Mixed Methods Study
Source: JMIR Res Protoc. 2017 Oct 16;6(10):e188. doi: 10.2196/resprot.7148 (PMC5662792; doi:10.2196/resprot.7148)
Supplement: Multimedia Appendix 1 [file resprot_v6i10e188_app1.pdf]

BP MONITOR: Semi-structured Interview Guide
[For Patients and their Family Caregivers]

Goal: To understand the feasibility and acceptability of self-monitoring blood pressure and medication self-titration
Setting: India (Bangalore), Malawi (Blantyre), Cameroon (Douala), Bangladesh (Dhaka), Pakistan (Gilgit), and Peru (Lima)
Number of focus groups*: 4 (Equal numbers of men and women, groups can be mixed also consider age group)
Number of participants: 6-9 (can include patients and their family caregivers)

*It is permissible to supplement the focus group interviews with individual interviews: up to 8 individual interviews (could be with patient and family)

INTERVIEW FLOW

A. Overall experience and attitudes
A1. Does high blood pressure worry you? Tell me why.

B. Measurement
B1. Do you get your blood pressure regularly checked?
Probe: If yes to B1, ASK: Where do you get it checked? Why do you get it checked? Are there any difficulties in getting your blood pressure checked?
Probe: If yes to B1: Does anyone in your family help you check your blood pressure?
Probe: If they say no to B1, ASK: Why? What stops from you getting it checked?

C. Treatment
C1. Are you taking medication for your blood pressure?
Probe: Are there things that make it difficult for you to take your medicine?
Optional Probe: How do you pay for your blood pressure medication?

D. Patient-physician relationship
D1. When your doctor gives you advice about high blood pressure or prescribes medicine for your high blood pressure, do you ever consult other people, family, friends, or other doctors, before following the doctor’s advice?

E. General description of BP MONITOR Intervention (develop medication plan with doctor, get home monitor, self-monitor, record readings, and titrate medicine)
E1. Would you be interested in participating in such a program/intervention?
Probe - Why? How do you think this could benefit you? Is there anything that worries you?

Final Thoughts
F1. What would make life better for people with high blood pressure?
