# Supplementary material for: Home Blood Pressure Management Intervention in Low- to Middle-Income Countries: Protocol for a Mixed Methods Study
Source: JMIR Res Protoc. 2017 Oct 16;6(10):e188. doi: 10.2196/resprot.7148 (PMC5662792; doi:10.2196/resprot.7148)
Supplement: Multimedia Appendix 2 [file resprot_v6i10e188_app2.pdf]

BP MONITOR: Semi-structured Interview Guide
[For Health Care Providers]

Goal: To understand the feasibility and acceptability of self-monitoring blood pressure and medication self-titration from the perspective of health care providers
Setting: India (Bangalore), Malawi (Blantyre), Cameroon (Douala), Bangladesh (Dhaka), Pakistan (Gilgit), and Peru (Lima)
Number of individual interviews*: 8-10 at each of 6 sites
Participants: Health care providers who diagnose, treat and care for patients with high blood pressure regularly.
*Sample should result in equal numbers of men and women if possible. Also consider age, type of provider (e.g., physician, nurse, community health worker, specialty and location of practice.)

INTERVIEW

A. Overall experience and attitudes
A1. How big a problem is high blood pressure in your patient population? Tell me more about that.

B. Measurement
B1. Do you ever have patients monitor their blood pressure at home or outside the clinic?
Probe: If yes to B1, ASK: What do you ask them to do? Why do you ask them to do this?
Probe: If they say no to B1, ASK: Why not?

C. Treatment
C1. Do you think most of your patients with high blood pressure follow the instructions/advice you give them? *Can probe more specifically*: about their medicine or about lifestyle?
Probe: Those who don't, what do you think are the barriers?
Alternative wording: What makes it difficult for patients to take their blood pressure medicine?

D. Patient-physician relationship
D1. Tell me how you see your role as a physician or {other health care provider} in helping patients with high blood pressure?
Alternative wording: What is the role of the clinician/health care team in blood pressure management?
D2. From your experience, what do you think is the role of the patient in blood pressure management?
D3. From your experience, how do family members help/support patients with blood pressure management?

E. General description of BP MONITOR Intervention (develop medication plan with doctor, get home monitor, self-monitor, record readings, and titrate medicine)
E1. Would you be interested in you and your patients participating in such a program/intervention?
Probe - Why? How do you think this could benefit you? Is there anything that worries you?
Probe: How do you think this could benefit your patients? Is there anything that worries you?

Final Thoughts
F1. What would make life better for people with high blood pressure?
F2. What would make it easier for you to take care of patients with high blood pressure?
